# *FOXP2* expression and gray matter density in the male brains of patients with schizophrenia

**DOI:** 10.1007/s11682-020-00339-x

**Published:** 2020-07-30

**Authors:** Julio Sanjuán, Xochitl Helga Castro-Martínez, Gracián García-Martí, Javier González-Fernández, Roberto Sanz-Requena, Josep María Haro, J. Javier Meana, Luis Martí-Bonmatí, Juan Nacher, Noelia Sebastiá-Ortega, Javier Gilabert-Juan, María Dolores Moltó

**Affiliations:** 1 Spanish National Network for Research in Mental Health CIBERSAM, Valencia, Spain; 2 Unit of Psychiatry, University of Valencia, Valencia, Spain; 3 INCLIVA Biomedical Research Institute, Fundación Investigación Hospital Clínico de Valencia, Valencia, Spain; 4 Department of Genetics, University of Valencia, Valencia, Spain; 5 Laboratorio de Genómica de Enfermedades Psiquiátricas y Neurodegenerativas, INMEGEN, Ciudad de México, México; 6 Biomedical Engineering Unit / Radiology Department, Quirónsalud Hospital, Valencia, Spain; 7 Parc Sanitari Sant Joan de Déu, Fundació Sant Joan de Deu, Barcelona, Spain; 8 Department of Pharmacology, Universidad del País Vasco/Euskal Herriko Unibertsitatea UPV/EHU, Leioa, Spain; 9 Neurobiology Unit, Cell Biology Department, Interdisciplinary Research Structure for Biotechnology and Biomedicine (BIOTECMED), Universitat de València, Valencia, Spain; 10 Department of Genetics, Universitat de València, Dr. Moliner 50, 46100 Burjassot, Valencia Spain

**Keywords:** *FOXP2*, Schizophrenia, Gray matter, Magnetic resonance imaging, Male

## Abstract

Common genetic variants of *FOXP2* may contribute to schizophrenia vulnerability, but controversial results have been reported for this proposal. Here we evaluated the potential impact of the common *FOXP2* rs2396753 polymorphism in schizophrenia. It was previously reported to be part of a risk haplotype for this disease and to have significant effects on gray matter concentration in the patients. We undertook the first examination into whether rs2396753 affects the brain expression of *FOXP2* and a replication study of earlier neuroimaging findings of the influence of this genetic variant on brain structure. *FOXP2* expression levels were measured in postmortem prefrontal cortex samples of 84 male subjects (48 patients and 36 controls) from the CIBERSAM Brain and the Stanley Foundation Array Collections. High-resolution anatomical magnetic resonance imaging was performed on 79 male subjects (61 patients, 18 controls) using optimized voxel-based morphometry. We found differences in *FOXP2* expression and brain morphometry depending on the rs2396753, relating low *FOXP2* mRNA levels with reduction of gray matter density. We detected an interaction between rs2396753 and the clinical groups, showing that heterozygous patients for this polymorphism have gray matter density decrease and low *FOXP2* expression comparing with the heterozygous controls. This study shows the importance of independent replication of neuroimaging genetic studies of *FOXP2* as a candidate gene in schizophrenia. Furthermore, our results suggest that the *FOXP2* rs2396753 affects mRNA levels, thus providing new knowledge about its significance as a potential susceptibility polymorphism in schizophrenia.

**Electronic supplementary material:**

The online version of this article (10.1007/s11682-020-00339-x) contains supplementary material, which is available to authorized users.

## Introduction

The *forkhead box P2* (*FOXP2)* gene was identified as the first gene involved in the development of speech and language (Lai et al. 2001). Rare *FOXP2* disruptions were reported as the causative mutations of severe language conditions (reviewed in Graham &Fisher 2015). Magnetic resonance imaging (MRI) studies in subjects carrying *FOXP2* mutations showed structural and functional abnormalities in language-related brain areas (Liégeois et al. 2016; Watkins et al. 2002). Agreeing, the *FOXP2* expression pattern is particularly relevant in the cortex, basal ganglia and other brain areas related to this human trait (Lai et al. 2003; Reimers-Kipping et al. 2011; Takahashi et al. 2008).

Language impairment and speech disorganization are core phenomenological characteristics of patients with schizophrenia; deficits in the neural organization of language were proposed to affect these patients (DeLisi 2001). This observation has led to the suggestion of *FOXP2* as a potential candidate gene for schizophrenia vulnerability. In addition, common genetic variations in *FOXP2* have been proposed to contribute to the etiology of this disease (Sanjuan et al. 2013). In a preliminary study, we found that the common *FOXP2* rs2396753 single nucleotide polymorphism (SNP) might be involved in language disorder vulnerability, including thought disorders and auditory hallucinations in schizophrenia (Sanjuan et al. 2006). These symptoms are related with structural, functional and connectivity alterations in brain pathways for language processing (Li et al. 2009). Using a larger sample of Spanish descent patients, we identified rs2396753 as part of a risk haplotype in schizophrenia (Tolosa et al. 2010). The involvement of *FOXP2* in schizophrenia vulnerability was further confirmed in a large sample of the Chinese Han population (Li et al. 2013). Other authors studying a sample of this population suggested that *FOXP2* might play a role in enhancing the vulnerability to psychotic symptoms in schizophrenia (Rao et al. 2017). In addition, McCarthy-Jones et al. (2014) found an interaction between a *FOXP2* SNP and parental child abuse, predicting susceptibility to psychotic auditory hallucinations. Interestingly, Spaniel and col. (2011) identified an association between the *FOXP2* rs2396753 and gray matter (GM) concentration changes in schizophrenia patients. Recently, a common *FOXP2* SNP was nominally associated with phonemic verbal fluency in the Western Australian Family Study of Schizophrenia (McCarthy et al. 2019).

Genetic variation in *FOXP2* has also been associated with speech and reading related phenotypes in healthy individuals. It includes differences in activation of the left frontal cortex during a reading task (Pinel et al. 2012), lateralization of speech perception (Ocklenburg et al. 2013), differences in inner speech and speech fluency (Crespi et al. 2017), semantic fluency (Mozzi et al. 2017) and auditory-motor integration for vocal pitch regulation (Zhang et al. 2018). Collectively, all these findings suggest that common polymorphisms of *FOXP2* may be involved in language related phenotypes in the general population and thereby may contribute to psychopathology and language impairment in neuropsychiatric disorders as schizophrenia. Nonetheless, controversial results were reported about associations between common variation of *FOXP2* and language in both clinical (McCarthy et al. 2019; Yin et al. 2018) and healthy subjects (Mueller et al. 2016). In addition, the possible influence of these variants in brain structures in the general population has also been questioned (Hoogman et al. 2014).

Here, we evaluated whether common polymorphisms of *FOXP2* influence brain structure in schizophrenia. We undertook the first examination into whether rs2396753 affects the brain expression of *FOXP2*. Second, we performed a replication study of earlier MRI findings of the influence of this SNP on brain structure (Spaniel et al. 2011), using a larger and more homogenous sample. As far as we know, this is the first study that combined structural MRI and brain gene expression analysis to search for the role of *FOXP2* in the schizophrenia vulnerability.

## Methods

### Experimental design

We executed a double approach for examining the relevance of the common *FOXP2* rs2396753 in schizophrenia: *FOXP2* expression was measured in postmortem human brain tissues from the prefrontal cortex (PFC); MRI was performed on 79 unrelated subjects recruited as part of a larger research program on schizophrenia. Statistical power of this study is indicated in supplementary material.

### Brain tissue samples

PFC samples of schizophrenia patients (n = 25) and control subjects with no history of psychosis (n = 11) were obtained from the Brain Collections of the Spanish National Network for Research in Mental Health CIBERSAM. RNA samples from the PFC of schizophrenia patients (n = 23) and matched unaffected controls (n = 25) were obtained from the Array Collection of the Stanley Medical Research Institute. All donor patients met DSM-IV criteria for schizophrenia. Detailed description of these samples is shown in Table 1 and the supplementary material.

**Table 1:** Demographic and tissue parameters of the postmortem brain samples

CIBERSAM tissues	Patients	Controls	*p*
Gender, n	males, 25	males, 11	N/A
Age of death, years. Mean (SD)	63.84 (16.17)	71.73 (10.92)	0.15
PMI, hours. Mean (SD)	8.00 (10.08)	8.82 (4.94)	0.80
Brain pH. Mean (SD)	7.12 (0.15)	7.00 (0.20)	0.05
Cause of death	6 Respiratory disease, 12 other medical conditions, 7 suicide	2 Cardiac, 4 Pneumonia, 5 other medical conditions	N/A
STANLEY RNA	**Patients**	**Controls**	*p*
Gender, n	males, 23	males, 25	N/A
Age of death, years. Mean (SD)	41.35 (8.53)	45.08 (7.82)	0.12
PMI, hours. Mean (SD)	30.13 (16.09)	27.88 (12.33)	0.59
Brain pH. Mean (SD)	6.42 (0.26)	6.66 (0.24)	0.0017*
Cause of death	11 cardiac disease, 7 other medical conditions, 1 accident, 4 suicide	23 cardiac, 2 other medical conditions	N/A

Mean ± standard deviation (SD) are shown for each variable; PMI, postmortem interval; N/A, not applicable; *p* value obtained when comparing patients and controls using the unpaired *t* test; * p < 0.05

### Subjects

The patient group included 61 subjects with DSM-IV diagnosis for schizophrenia. All patients were assessed with the Positive and Negative Syndrome Scale (PANSS) (Kay et al. 1987; Peralta and Cuesta 1994) by a trained evaluator, who was blind to the genotyping and MRI results. Eighteen healthy control subjects were also studied. Both groups (Table 2) were matched by sex (all males), ethnic group (all Caucasian) and laterality (all right-handed). Detailed description of the inclusion criteria for the MRI study is shown in the supplementary material.

**Table 2:** Demographic and clinical data for subjects who underwent the morphometric MRI examination accounting for *FOXP2* rs2396753 genotypes (AA, AC and CC)

	Patients (n = 61)					Controls (n = 18) AA: 3; AC: 13; CC: 2	
	AA	AC	CC	Total	*(F, p)* ^a^	Total	*(F, p)* ^b^
N (%) all males	20 (32.8%)	27 (44.3%)	14 (23.0%)	61 (100%)	N/A	18 (100%)	N/A
Age, years. Mean (SD)	29.52 (9.20)	32.88 (10.97)	33.25 (9.50)	31.87 (10.06)	F = 0.80 p = 0.45	38.24 (7.89)	F = 6.09 p = 0.01*
TIV, cm^3^. Mean (SD)	1082.38 (86.54)	1096.83 (102.63)	1095.35 (99.06)	1091.75 (95.43)	F = 0.14 p = 0.87	1139.88 (82.55)	F = 3.74 p = 0.06
PANSS Total. Mean (SD)	61.88 (22.94)	68.86 (10.67)	58.92 (21.25)	64.24 (18.15)	F = 1.37 p = 0.26	N/A	N/A
PANSS Positive. Mean (SD)	15.44 (6.03)	16.64 (5.21)	14.75 (6.27)	15.80 (5.68)	F = 0.46 p = 0.63	N/A	N/A
PANSS Negative. Mean (SD)	15.19 (6.95)	18.64 (5.63)	14.33 (6.30)	16.50 (6.41)	F = 2.37 p = 0.11	N/A	N/A
PANSS General. Mean (SD)	31.88 (11.31)	33.59 (5.56)	29.83 (9.92)	31.98 (8.77)	F = 0.76 p = 0.47	N/A	N/A

Mean ± standard deviation (SD) are shown for each variable; TIV (Total Intracranial Volume); PANSS (Positive and Negative Syndrome Scale); N/A (not applicable); ^a^ statistics (*F* and *p* values) comparing AA, AC and CC genotypes in schizophrenia patients using one-way ANOVA test; ^b^ statistics (*F* and *p* values) comparing the clinical groups (patients with schizophrenia and healthy control subjects) using one-way ANOVA test. * p < 0.05

### Genotyping

Genomic DNA (gDNA) was extracted from peripheral blood leukocytes and from CIBERSAM PFC samples using the Puregene kit (Gentra Systems, Qiagen). The Array Collection gDNA was donated by the Stanley Medical Research Institute. Genotyping of *FOXP2* rs2396753 was performed at the Spanish Genotyping Center of Santiago de Compostela using the iPLEX Gold technology from Sequenom. For the CIBERSAM samples, genotyping was carried out as previously indicated (Tolosa et al. 2010).

### Reverse transcription quantitative PCR (RT-qPCR)

Total RNA from the CIBERSAM samples was extracted using the miRNeasy Mini Kit (Qiagen) according to the manufacturer’s procedure. RNA from the CIBERSAM and Stanley samples was converted into cDNA as previously described (Gilabert-Juan et al. 2015). Amplification was performed using the Step One Plus Real-Time PCR System on an Applied Biosystems 7700 (Applied Biosystems) and SYBR Green PCR master mix (Applied Biosystems). RT-qPCR analyses are further described in the supplementary material.

### Quantitative neuroimaging

#### Magnetic resonance acquisition

All subjects underwent an MRI examination on a 3T magnet (Philips Achieva, Best, The Netherlands). The acquisition protocol included a 3-dimensional spoiled gradient-echo pulse sequence (TE = 7.38 ms; TR = 13.18 ms; FA = 8º, NEX = 1, 160 slices, thickness = 1 mm, matrix = 256 × 256, FOV = 240 mm, voxel size = 0.90 × 0.90 × 1 mm).

#### Data processing

Statistical parametric mapping 8 (SPM8) software (SPM, Wellcome Institute, London, United Kingdom) and MATLAB R2015a (The MathWorks, Natick, MA, USA) were used to process the data. The raw images were evaluated quantitatively to detect outliers, which were considered as the images with a high degree of variability (above 3*standard deviations) from the mean. No images were identified as outliers.

The voxel-based morphometry (VBM8) and the Diffeomorphic Anatomical Registration Through Exponentiated Lie Algebra (DARTEL) method (Ashburner 2007) was used to normalize and to segment the original images. The GM and white matter (WM) maps obtained after segmentation were again registered to construct a more accurate diffeomorphic template. The original raw images were then warped to the DARTEL template using flow fields and spatial deformations. To preserve the total amount of tissue in the normalized images, a Jacobian transformation was applied. Finally, the warped and modulated GM images were smoothed by a 10 mm full width at half maximum Gaussian smoothing filter. This value was chosen considering the relatively small number of subjects in each genotype group, which requires a bigger smoothing kernel to improve signal-to-noise ratio (Shen and Sterr 2013). Detailed description of quantitative neuroimaging analysis is further indicated in supplementary material.

### Statistical analysis

Tissue features, demographic and clinical data of subjects and *FOXP2* expression were compared using ANOVA and t-test. Bonferroni test (Rice 1989) was applied for multiple-comparison correction. In all cases, values of *p* < 0.05 were considered statistically significant. Error bars represent standard deviation (SD).

In neuroimaging, three different statistical models were created and estimated according to the general linear model (Friston et al. 1994). The GM probability maps and the two nuisance variables, age and total intracranial volume (TIV), were included in these models. First, a two-sample t-test model was constructed to test for GM differences between patients and controls. These maps were then binarized (*assigning 1 to those voxels with T values greater than 0 and 0 to the rest*) and used as a restriction-mask in the later analyses. Second, an ANCOVA model was designed including only the rs2396753 genotype. This model was used to detect subtle anatomical GM differences related to the genotype considering all subjects. Third, a full-factorial ANCOVA model was tested to assess the GM differences between the subjects by accounting for two main factors: clinical group (patients versus controls) and rs2396753 genotype. An extent threshold filter was applied to consider only those clusters with the minimum number of contiguous voxels (k) as the expected number of voxels per cluster provided by SPM8. The statistical threshold was fixed at p < 0.05 with a family-wise error (FWE) rate correction for multiple comparisons at the cluster level.

## Results

### *FOXP2* expression in postmortem brain tissues

Table 3 shows the demographic and tissue-related variables of the brain samples grouped regarding the rs2396753 genotypes. These traits showed nonsignificant differences among the three genotypes in either the patients or controls. Nonsignificant differences were observed when comparing patients *versus* controls, except for the suicide events.

**Table 3:** Demographic and tissue characteristics of the postmortem brain samples accounting for clinical group (patients and controls) and rs2396753 genotypes of *FOXP2* (AA, AC and CC)

	Patients (n = 48)					Controls (n = 36)					
	AA	AC	CC	Total	(*F, p*) ^a^	AA	AC	CC	Total	(*F, p*) ^b^	*p* ^c^
N (%) all males	13(27.1%)	27(56.2%)	8(16.7%)	48(100%)	N/A	18(50.0%)	15(41.7%)	3(8.3%)	36(100%)	N/A	0.0866^d^
Age, years. Mean (SD)	47.38 (14.71)	55.56 (18.93)	53.88 (14.27)	53.06 (17.22)	F = 1.00 p = 0.38	53.72 (16.42)	50.53 (13.95)	63.67 (12.90)	53.22 (15.20)	F = 0.95 p = 0.40	0.96
PMI, hours. Mean (SD)	23.77 (15.54)	16.30 (17.91)	18.00 (18.00)	18.60 (17.26)	F = 0.82 p = 0.45	21.78 (13.90)	22.80 (12.70)	20.00 (23.52)	22.06 (13.80)	F = 0.06 p = 0.95	0.33
Brain pH. Mean (SD)	6.71 (0.34)	6.81 (0.41)	6.84 (0.52)	6.79 (0.41)	F = 0.34 p = 0.71	6.74 (0.30)	6.81 (0.25)	6.73 (0.31)	6.77 (0.28)	F = 0.27 p = 0.76	0.83
Number of suicide cases	2	5	4	11		0	0	0	0		

Mean ± standard deviation (SD) are shown for each variable, with the exception of suicide cases; PMI, postmortem interval; N/A, not applicable; ^a^ statistics (*F* and *p* values) comparing AA, AC and CC genotypes in schizophrenia patients using one-way ANOVA test; ^b^ statistics (*F* and *p* values) comparing AA, AC and CC genotypes in control subjects using one-way ANOVA test; ^c^ *p* value between total patients and total controls using unpaired *t*-test; ^d^ Genotype differences between patients and controls were compared using the chi-squared test, χ^2^ = 4.893, df = 2

**Fig. 1:**
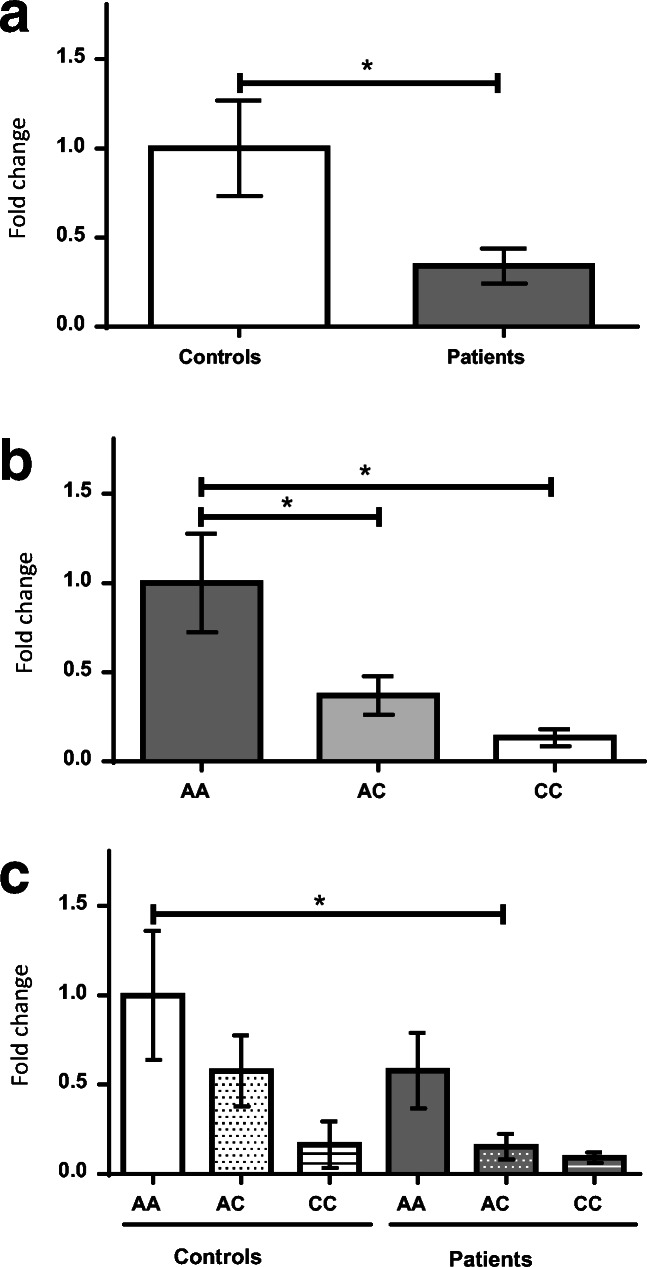
*FOXP2* expression in the prefrontal cortex of male donor subjects with schizophrenia versus controls (A); accounting for the *FOXP2* rs2396753 polymorphism in the total samples (B); taking into consideration the interaction between this polymorphism and the clinical groups (C). *FOXP2* expression is indicated as fold changes relative to the control group (A), AA genotype (B) and AA genotype of the control group (C) considering its expression as 1 in each case. Asterisk indicates the degree of significance (* p < 0.05) using an unpaired *t* test (A) and one-way ANOVA with Bonferroni multiple comparison tests (B and C). Error bars represent standard deviation

*FOXP2* expression was reduced in the PFC of the schizophrenia patients (*p* = 0.0121; Fig. 1A). In the whole sample, subjects carrying the AA genotype of rs2396753 showed higher *FOXP2* expression than those with the AC and CC genotypes (Fig. 1B; *p* = 0.0451 and *p* = 0.0281 after the Bonferroni correction, respectively). The AC genotype exhibited an intermediate expression between both homozygotes, although the differences were not significant in comparison to the expression of the CC genotype (Fig. 1B). Overall, these results indicated a lower expression of the C allele in respect to the A allele of this SNP. When comparing the *FOXP2* expression in each clinical group, we also observed the differential expression of both alleles (Fig. 1C). Interestingly, heterozygous samples showed different *FOXP2* expression levels in the patients compared to the controls, although this change was nonsignificant. Significance was maintained between the controls with the AA genotype and the patients with the AC genotype after the Bonferroni correction (*p* = 0.0288; Fig. 1C).

### Magnetic resonance imaging in patients with schizophrenia and control subjects

No differences were found between patients and controls for the TIV (Table 2). However, there was a significant difference in age between the two clinical groups (*p =* 0.01); thus, age was included as a covariate in subsequent statistical analysis. In the patient group, no differences were detected for age, TIV or PANSS scores regarding the rs2396753 (Table 2).

**Fig. 2:**
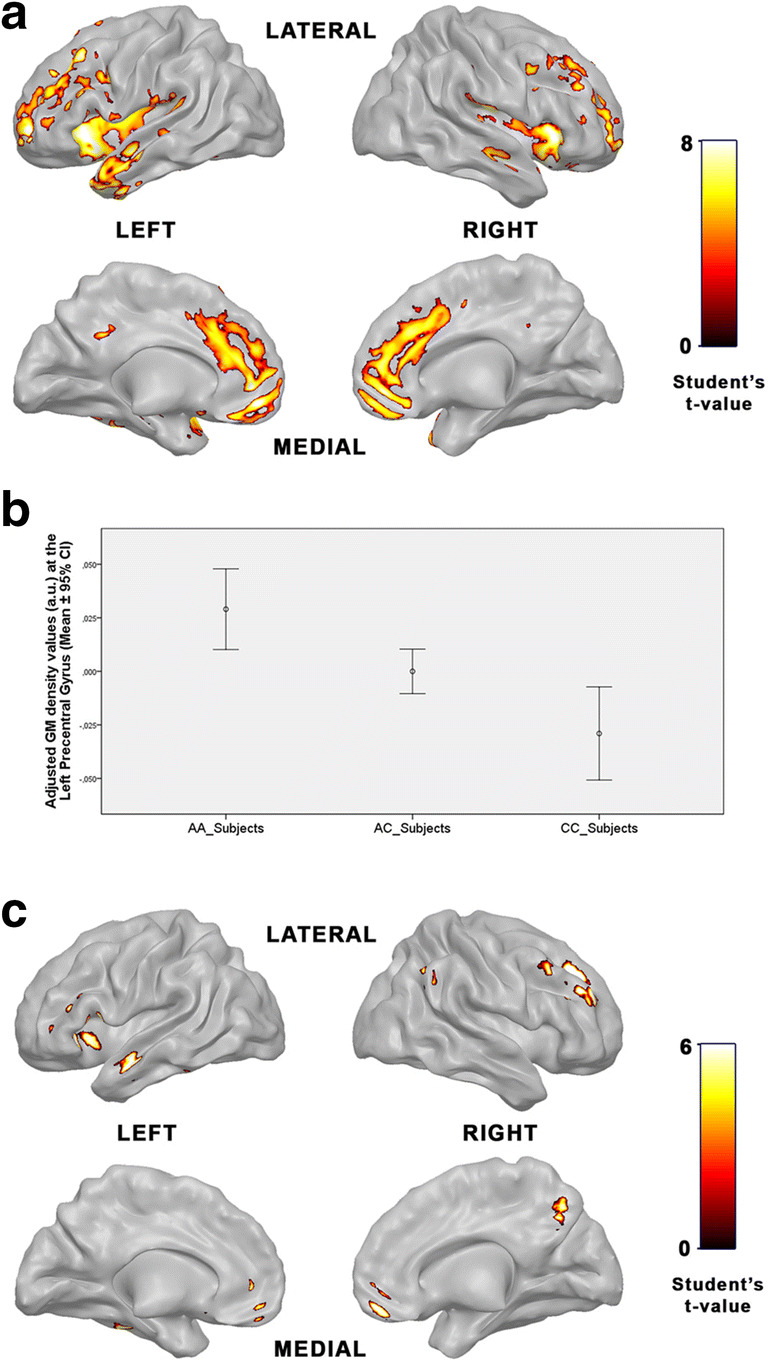
Brain imaging data of the male subjects who underwent the MRI examination. Areas with GM density reductions in patients with schizophrenia compared with control subjects, p < 0.05 FWE, k = 65 (A); Distribution of the adjusted GM density values in the left precentral gyrus (-36, 3, 41) concerning the rs2396753 genotypes of *FOXP2* in the overall sample (patients and controls).These values are shown as arbitrary units (a.u.). Error bars represent standard deviation (B); Areas with GM density reduction in AC heterozygote patients compared with AC heterozygote controls in terms of the rs2396753, p < 0.05, corrected at voxel level, k = 25 (C)

We found a significant GM density reduction (*p* < 0.05 FWE, k = 65) in patients with schizophrenia versus controls after correcting for the effects of age (F = 6.09; *p* = 0.02) and TIV (F = 3.74; *p* = 0.06). This reduction was mainly located in insular, temporal, frontal and cingulate areas (Fig. 2A; Supplementary Table 1). Nonsignificant increases in GM were found in patients compared to controls.

In the whole sample, subjects with the CC genotype showed a significant decrease in the GM density in respect to the AA and the AC genotypes in the left precentral gyrus. There were no differences in age (F = 0.82; *p* = 0.44) or TIV (F = 0.65; *p* = 0.53) between genotype groups (Fig. 2B; Supplementary Table 2). These results indicated that the C allele is associated with lower GM density in this brain area in males. This finding is consistent with the intermediate values of this trait showed by subjects carrying the AC genotype (Fig. 2B).

Next, GM differences were analyzed taken into account the interaction of the *FOXP2* rs2396753 and the clinical group. Only the comparisons with more than five subjects in each genotype group were considered to have reliable results. Due to the reduction of samples per group (AC patients vs. AC controls), and for exploratory purposes, we applied the FWE correction for multiple comparisons only at voxel level (not at cluster level) in this comparison. We found a significant (*p* < 0.05, corrected at voxel level, k = 25) GM density reduction in the AC patients when compared with AC controls. Neither age (F = 1.54; *p* = 0.22) nor TIV (F = 2.48; *p* = 0.12) showed significant differences between groups. The affected areas were mainly the frontal, temporal and parietal regions (Fig. 2C; Supplementary Table 3).

## Discussion

In this study, we found a clear concordance between *FOXP2* expression and GM density in the brain regarding rs2396753. Our results suggest that low *FOXP2* expression is linked to GM density reduction in frontal brain regions in males and that variations in *FOXP2* expression in such regions may provoke changes in GM density.

Several studies have related the *FOXP2* rs2396753 with schizophrenia. We previously observed a higher frequency of the C allele in patients than in controls; this allele was found to be part of a risk haplotype (Sanjuan et al. 2006). We later replicated this finding using a larger sample of Spanish descend subjects (Tolosa et al. 2010). In addition, the rs2396753 C allele was correlated with reductions in GM density in several brain regions of schizophrenia patients, including the dorsolateral prefrontal cortex (Spaniel et al. 2011). The GM deficits were more extensive in the AC schizophrenia patients, affecting the PFC, the anterior and median cingulate and the paracingulate gyri, which suggested that reduction in the GM concentration in schizophrenia may be driven by the C allele. Our results replicated these findings and further showed differences in *FOXP2* expression depending on rs2396753, linking for the first time low *FOXP2* expression to GM density reductions.

Interestingly, the *FOXP2* rs2396753 polymorphism was also linked to brain activity in language-related areas in healthy adults. It was associated with performance of the auditory dichotic listening task in a sample of Caucasian descents (Ocklenburg et al. 2013). This result suggested that common variations in *FOXP2* might explain interindividual variability in language lateralization, a feature that is reduced in schizophrenia (Bleich-Cohen et al. 2012; Chou et al. 2017; Ocklenburg et al. 2015). Interindividual variability in the activation of the left frontal cortex in healthy subjects was also correlated with two *FOXP2* SNPs in high linkage disequilibrium with rs2396753 (Pinel et al. 2012), suggesting that there may be a continuum between normal and pathological conditions in which *FOXP2* might be involved. Nevertheless, the effect of common genetic variants of *FOXP2* on the variability in brain morphometry is a matter of debate. Hoogman et al. (2014) did not find association between common variants in *FOXP2* and brain structure variability in a large sample of the general population. It is likely that common variations in this gene could contribute differentially to variability in neuroanatomy depending on sex and clinical condition. In this vein, *Foxp2* expression was shown to have a sex-specific spatial and temporal pattern in the developing mammalian brain, which is influenced by androgens (Bowers et al. 2014; Fröhlich et al. 2017). Sex differences in *FOXP2* expression were also reported in human cortex samples from non-diseased donors (Bowers et al. 2013). Thus, sex should be taken into account when analyzing the effects of *FOXP2* polymorphisms.

The rs2396753 is an intronic SNP located between exons 2b and 3 of *FOXP2*. Therefore, it might control the *FOXP2* expression level in specific cells or tissues. Our results suggest that rs2396753, or a causal variant in linkage disequilibrium, participates in *FOXP2* regulation, because the *FOXP2* mRNA levels are higher in the AA than the CC genotypes (Fig. 1B). Importantly, the A allele shows putative binding sites for transcription factors belonging to the V$ETSF, V$GABF and V$ZF35 families, while they are absent in the C allele. However, this allele has a putative binding site for V$STAF transcription factors (SNPinspector 2.4 from Genomatix Software Suite v3.9; Supplementary Table 4). The V$ETSF family contains members that are positive regulators of transcription (Oikawa and Yamada 2003), meanwhile members of the V$STAF family function as transcriptional repressors (Zheng and Yang 2004). Accordingly, we can consider that transcription factors from these families might contribute to the different expression levels of the A and C alleles. Detailed molecular analyses are needed to uncover the regulatory process implicated in the expression differences of both alleles.

We found an interesting result concerning the AC genotype of the *FOXP2* rs2396753. Epigenetic factors, including medication, might contribute to the different expression level of *FOXP2* between patients and controls carrying this genotype. Differences of this type were also described for the T102C polymorphism of the *HTR2A* gene (Abdolmaleky et al. 2011). In this case, the C allele showed higher expression than that of the T allele, being more evident in controls than in patients with schizophrenia and bipolar disease. The authors found that the *HTR2A* expression level was inversely correlated with the degree of DNA methylation at the *HTR2A* promoter. Whatever the cause, the lower expression of *FOXP2* in heterozygous schizophrenia patients than heterozygous controls could explain the reduced expression of this gene that we found in PFC of schizophrenia patients (Fig. 1A).

The underlying molecular mechanism by which *FOXP2* may be implicated in the decrease in GM density in schizophrenia patients is unknown and merits further research. Abnormalities in GM volumes in cortical regions in schizophrenia were proposed to be caused by impairments in embryonic neuronal migration (Jamadar et al. 2011). Interestingly, several studies described the role of *FoxP2* in neuronal recruitment in Area X, which is associated with vocal learning in zebra finches (Haesler et al. 2007; Rochefort et al. 2007). *FOXP2* encodes a transcription factor that regulates multiple downstream targets implicated in development, patterning and function of the nervous system, including key players of neural migration (French and Fisher 2014; Vernes et al. 2007, 2011).

Some limitations should be considered in this study. The small number of controls with the AA and CC genotypes in our subject sample that underwent MRI prevented comparison of the effect of these genotypes on GM in schizophrenia patients versus controls. Additionally we assume that there may be a potential bias because age in the tissue sample was greater than in the neuroimaging dataset. Finally, differences in *FOXP2* expression may be found in other brain regions where this gene is expressed.

In conclusion, we provide a plausible explanation of the reduction in GM density observed in schizophrenia patients through differential expression of *FOXP2*. Our results supported the previous view that posited a link between the genetic variation of *FOXP2* and brain morphometry in schizophrenia.

## Electronic supplementary material

ESM 1 (PDF 236 kb)
