# Supplementary material for: FOXP2 expression and gray matter density in the male brains of patients with schizophrenia
Source: Brain Imaging Behav. 2020 Jul 30;15(3):1403–11. doi: 10.1007/s11682-020-00339-x (PMC8286223; doi:10.1007/s11682-020-00339-x)
Supplement: Supplementary file 1 — (PDF 236 kb) [file 11682_2020_339_MOESM1_ESM.pdf]

FOXP2 expression and gray matter density in the male brains of patients with schizophrenia

Julio Sanjuán[1,2,3], Xochitl Helga Castro-Martínez[4,5], Gracián García-Martí[1,6], Javier González-Fernández[4], Roberto Sanz-Requena[6], Josep María Haro[1,7], J. Javier Meana[1,8], Luis Martí-Bonmatí[6], Juan Nacher [1,3,9], Noelia Sebastiá-Ortega[1,4], Javier Gilabert-Juan[1,3,4,9,*] (ORCID: 0000-0001-5187-3942), María Dolores Moltó [1,3,4,*] (ORCID: 0000-0001-5219-2022)

[1] Spanish National Network for Research in Mental Health CIBERSAM, Spain.

[2] Unit of Psychiatry, University of Valencia, Valencia, Spain.

[3] INCLIVA Biomedical Research Institute, Fundación Investigación Hospital Clínico de Valencia, Valencia, Spain.

[4] Department of Genetics, University of Valencia, Valencia, Spain.

[5] Laboratorio de Genómica de Enfermedades Psiquiátricas y Neurodegenerativas, INMEGEN, Ciudad de México, México.

[6] Biomedical Engineering Unit / Radiology Department, Quirónsalud Hospital, Valencia, Spain.

[7] Parc Sanitari Sant Joan de Déu, Fundació Sant Joan de Deu, Barcelona, Spain.

[8] Departamento de Farmacología, Universidad del País Vasco/Euskal Herriko Unibertsitatea UPV/EHU, Spain.

[9] Neurobiology Unit, Cell Biology Department, Interdisciplinary Research Structure for Biotechnology and Biomedicine (BIOTECMED), Universitat de València, Valencia, Spain.

* Corresponding authors at Department of Genetics, Universitat de València, Dr. Moliner 50, Burjassot, 46100 Valencia, Spain:

Javier Gilabert-Juan, e-mail address: Javier.gilabert@uv.es; telephone number: 34-963543400

María Dolores Moltó, e-mail address: dmolto@uv.es; telephone number: 34-963543400

Methods

Statistical power of the study

The statistical power obtained for the postmortem samples was 0.65 using the ClinCalc calculator (Rosner, 2011) at https://clincalc.com/Stats/Power.aspx, and 0.58 using the G*Power version 3.0.10 (Faul et al., 2007). When the sample was divided in genotypes, the statistical power was reduced to 0.34 (G*Power) due to the small number of individuals in the "CC" genotype.

For neuroimaging data, we applied the G*Power version 3.0.10 (Faul et al., 2007).Using a 10 mm sphere on each cluster of the resulting parametric maps, we have obtained an averaged statistical power of 0.73 for the comparison between patients and controls. Regarding the imaging analysis between genotype groups, the averaged statistical power falls to 0.54 mainly due to the small number of subjects in each group.

Brain tissue samples

All samples belonged to Caucasian male donors with the exception of one Hispanic case in the schizophrenia patients group.

Subjects

None of the participants had previous electroconvulsive therapy or severe head trauma, present or past criteria for drug abuse (except for tobacco and cannabis), or any standard contraindications to the MRI examination.

RT-qPCR

RNA extraction from the CIBERSAM samples was carried out in RNAse-free environment. The purified total RNA was eluted in RNAse-free water and stored at -80ºC. Nucleic acid concentration and purity were measured at 260 nm and 260nm/280nm respectively by spectrophotometry (Eppendorf BioPhotometer plus), obtaining purity values of 2. The integrity of RNA was further assessed calculating the RNA Integrity Number (RIN) using an Agilent 2100 Bioanalyzer. These samples showed RIN values between 4.0 and 8.0. Regarding

the RNA from the Stanley array collection, poor RNA quality is an exclusion criteria for all specimens included in the collection (http://www.stanleyresearch.org/brain-research/).

Primers for the transcript amplification of *FOXP2* were designed to amplify a 116 bp product between exons 9 and 11 to avoid potential gDNA contamination (Tolosa et al. 2010). *Beta-actin* (*ACTB*) was used to normalize the expression levels of *FOXP2* since *ACTB* was the most stable housekeeping gene analyzed in our samples using the free Norm Finder software (http://www.mdl.dk/publicationsnormfinder.htm), previously developed and validated (Andersen et al. 2004). Each sample was run in triplicate. Following a 95 ºC denaturation for 3 min, the reactions were cycled 50 times with a 95 ºC denaturation for 30 s, a 57 ºC annealing step for 30 s and a 72 ºC extension for 30 s. A melt curve was obtained to calculate primers efficiency for the target and the reference gene. We obtained efficiency values of 94.961% for *FOXP2* and 95.691% for *ACTB* that are similar values and are within the desired amplification efficiencies range from 90% to 110%. Relative quantification of the gene expression was conducted using the comparative threshold Ct method according to the 2_ΔΔCt method (Pfaffl 2001), where ΔΔCt = (Ct, target gene – Ct, reference gene) exp. group – (Ct, target gene – Ct, reference gene) control group.

Quantitative neuroimaging

After acquisition, the MR images were qualitatively reviewed by a radiologist and a computer engineer, who were both blind to clinical and genetic data, to ensure data quality. The images were then anonymized for analysis and post-processing.

To reduce the bias derived from using predefined templates (Shen et al. 2007), a custom set of tissue-templates was created that included all subjects. First, the raw images were normalized to the standard Montreal Neurological Institute of McGill University Health Center (MNI152) template using affine transformations. Second, the normalized MR images were segmented, averaged and smoothed using a 3D Gaussian smoothing kernel with an 8 mm FWHM to create whole brain, GM and WM tissue templates. Next, the original MR volumes of

each participant were normalized and segmented using the custom template dataset as a reference. The final voxel size of the normalized images was fixed to 1.5x1.5x1.5 mm.

References

Andersen, C.L., Jensen, J.L., & Ørntoft, T.F. (2004). Normalization of Real-Time Quantitative Reverse Transcription-PCR Data: A Model-Based Variance Estimation Approach to Identify Genes Suited for Normalization, Applied to Bladder and Colon Cancer Data Sets. *Cancer Research, 64*(15), 5245-5250. https://doi.org/10.1158/0008-5472.CAN-04-0496

Faul, F., Erdfelder, E., Lang, A.-G., and Buchner, A. (2007). G*Power 3: A flexible statistical power analysis program for the social, behavioral, and biomedical sciences. *Behavior Research Methods*, 39, 175-191. https://doi.org/10.3758/bf03193146

Pfaffl, M.W. (2001). A new mathematical model for relative quantification in real-time RT-PCR. *Nucleic Acids Research, 29*(9), e45. https://doi.org/ 10.1093/nar/29.9.e45

Rosner B. Fundamentals of Biostatistics. 7th ed. Boston, MA: Brooks/Cole; 2011.

Shen, S., Szameitat, A. J., & Sterr, A. (2007). VBM lesion detection depends on the normalization template: a study using simulated atrophy. *Magnetic Resonance Imaging, 25*(10), 1385-1396. https://doi.org/10.1016/j.mri.2007.03.025

Tolosa, A., Sanjuán, J., Dagnall, A. M., Moltó, M. D., Herrero, N., & de Frutos, R. (2010). FOXP2 gene and language impairment in schizophrenia: association and epigenetic studies. *BMC Medical Genetics, 11,* 114-121. https://doi.org/10.1186/1471-2350-11-114

Supplementary Table 1. Areas with GM density reduction in patients with schizophrenia compared with control subjects ($p<0.05$ FWE, $k = 65$).

Student-t value	Coordinate	Label	Right / Left	Brodmann area
7.67	(-27, 53, -3)	Insula	Left	32
6.81	(36, 27, 0)	Insula	Right	48
6.13	(-47, -56, -15)	Inferior Temporal Gyrus	Left	20
6.05	(39, 20, -33)	Middle Temporal Gyrus	Right	38
5.96	(41, 8, 36)	Precentral Gyrus	Right	44
5.74	(-24, -14, -18)	Hippocampus	Left	20
5.53	(-5, -45, 35)	Middle Cingulum	Left	23
5.44	(-20, 21, 48)	Superior Frontal Gyrus	Left	08
5.36	(-53, -32, -5)	Middle Temporal Gyrus	Left	21

Supplementary Table 2. LSD Post hoc test over the GM density values at the Left Precentral Gyrus (-36, 3, 41) between genotype.

Comparison	LSD Post hoc test
$GM_{AC\_Subjects} < GM_{AA\_Subjects}$	MS=0.029; p=0.004 **
$GM_{CC\_Subjects} < GM_{AA\_Subjects}$	MS=-0.058; p<0.000 ***
$GM_{CC\_Subjects} < GM_{AC\_Subjects}$	MS=-0.029; p=0.010 *

*p<0.05, **p<0.005, ***p<0.0005

Supplementary Table 3. Areas with GM density reduction in AC Patients compared with AC_Controls ($p<0.05$, corrected at voxel level, $k = 25$).

Student-t value	Coordinate	Label	Right / Left	Brodmann area
4.74	(21, 35, 38)	Superior Frontal Gyrus	Right	09
4.43	(-17, 20, 48)	Superior Frontal Gyrus	Left	08
4.36	(-39, 36, -3)	Insula	Left	47
4.13	(-50, -9, -20)	Middle Temporal Gyrus	Left	21
4.05	(35, 44, 24)	Middle Frontal Gyrus	Right	46
3.96	(53, -54, 35)	Inferior Parietal Gyrus	Right	39
3.86	(-41, 26, 17)	Inferior Frontal Gyrus	Left	48
3.77	(-45, -36, -23)	Inferior Temporal Gyrus	Left	20
3.68	(3, 48, -12)	Middle Orbitofrontal Gyrus	Left	11
3.66	(-53, 12, 8)	Inferior Frontal Opercular Gyrus	Left	48
3.45	(41, 20, 42)	Middle Frontal Gyrus	Right	46

Supplementary Table 4. Putative binding sites for transcription factors (TF) affected by the *FOXP2* rs2396753 polymorphism.

Family of TF	Position	DNA Strand	Allele A	Allele C	Description
V$ETSF	491-511	+	Present	Absent	Human and murine ETS1 factors
V$GABF	491-515	+			GAGA-Box
V$ZF35	495-507	+			Zinc finger protein ZNF35
V$STAF	492-522	-	Absent	Present	Zinc finger protein 143

[gnl|dbSNP|rs2396753 rs=2396753|pos=501|len=1001|taxid=9606|mol="genomic"|class=1|alleles="A/C"|build=147|suspect=?|GMAF=C:5008:0.324281]
